# Extracellular sphingomyelinase activity impairs TNF-α-induced endothelial cell death via ADAM17 activation and TNF receptor 1 shedding

**DOI:** 10.18632/oncotarget.19983

**Published:** 2017-08-07

**Authors:** Anselm Sommer, Marie Düppe, Lena Baumecker, Felix Kordowski, Joscha Büch, Johaiber Fuchslocher Chico, Jürgen Fritsch, Stefan Schütze, Dieter Adam, Maria Sperrhacke, Sucharit Bhakdi, Karina Reiss

**Affiliations:** ^1^ Department of Dermatology, University of Kiel, 24105 Kiel, Germany; ^2^ Institute of Immunology, University of Kiel, 24105 Kiel, Germany

**Keywords:** sphingomyelinase, ADAM17, apoptosis, TNF-α, TNFR1

## Abstract

ADAM17, a prominent member of the “Disintegrin and Metalloproteinase” (ADAM) family, is an important regulator of endothelial cell proliferation and cell survival. The protease controls vital cellular functions through cleavage of growth factors, cytokines and their receptors including transforming growth factor-alpha (TGF-α), tumor necrosis factor-alpha (TNF-α) and TNF-α receptor 1 (TNFR1). TNF-α is the major inducer of endothelial cell death in cardiovascular diseases. The latter are also characterized by elevated plasma and tissue levels of extracellular sphingomyelinase (SMase). Whether the SMase affects ADAM activity and thus endothelial cell function has not been addressed to date. Here, we analyzed the effect of SMase on ADAM17-mediated shedding in COS7 cells and in human umbilical vein endothelial cells (HUVECs). Exposure to SMase significantly increased ADAM17-mediated release of alkaline-phosphatase (AP)-tagged TGF-α in COS7 cells and shedding of endogenously expressed TNFR1 in HUVECs. We previously presented evidence that surface exposure of phosphatidylserine (PS) is pivotal for ADAM17 to exert sheddase function. We found that SMase treatment led to PS externalization in both cell types. Transient non-apoptotic PS exposure is often mediated by Ca^2+^-dependent phospholipid scramblases. Accordingly, the Ca^2+^-chelator EGTA markedly reduced the breakdown of phospholipid asymmetry and shedding of TGF-α and TNFR1. Moreover, sheddase activity was significantly diminished in the presence of the competing PS-headgroup OPLS. SMase-stimulated TNFR1 shedding strikingly diminished TNF-α-induced signalling cascades and endothelial cell death. Taken together, our data suggest that SMase activity might act as protective factor for endothelial cells in cardiovascular diseases.

## INTRODUCTION

The discovery of ceramide as a central player in the sphingolipid signaling network opened a new area in biology, providing links to fundamental events involved in cell death and differentiation, oncogenesis, and inflammation [1–4]. Ceramide generation occurs through cleavage of sphingomyelin, which is located mainly in the outer leaflet of the plasma membrane. This process can be mediated by sphingomyelinases (SMases), preferentially active at either acid (aSMase) or neutral (nSMase) pH [5]. Dependent on posttranslational modification, aSMase is trafficked either to the lysosome or secreted extracellularly. Elevated serum levels of soluble aSMase have been reported in pathological states including atherosclerosis, type II diabetes, sepsis, chronic heart failure, and hyercytokinemia [5]. In cell culture, aSMase is secreted by various cell types including human vascular endothelial cells [6]. However, hardly anything is known about the function of soluble aSMase in the extracellular environment, where neutral pH bars its activity [7]. Neutral sphingomyelinase (nSMase) is located intracellularly and thus does not reach its substrate in the unperturbed cell [5, 8].

Yet, ceramide generation by both aSMase and nSMase has been implicated as an important event occurring in apoptotic cells [5, 7, 9–13]. The relevance thereof is a subject of continuing research. Ceramide generation *per se* does not promote cell death, as has been demonstrated in experiments employing bacterial sphingomyelinase from *Staphylococcus* (*S.*) *aureus* that is active at neutral pH. Application of the soluble enzyme at high concentrations that cause massive ceramide generation does not lead to death of resting lymphocytes, fibroblasts or endothelial cells [14, 15]. The possibility that ceramide might subserve a hitherto undescribed function in the context of sphingolipid-derived cell signaling events appears likely.

Published reports on the effects of ceramide on the organization of membrane lipids provided us with the decisive lead for the present work. Investigations conducted with erythrocytes and platelets disclosed that ceramide disrupts membrane phospholipid asymmetry, leading to exposure of phosphatidylserine (PS) at the external membrane face [16, 17]. These seminal studies, which capitalized on the use of bacterial sphingomyelinase as the generator of ceramide, did not extend to nucleated cells. In the latter, we recently discerned that transient PS exposure occurred in response to diverse major signaling events including Ca^2+^-elevation, purinergic receptor activation, and protein kinase C activation [18, 19]. PS exposure appeared to be the decisive event leading to manifestation of the sheddase function of ADAM17, predominant member of the family of Zn^2+^-dependent transmembrane metalloproteinases. ADAM17 fulfills multifaceted, vital roles in cell biology by liberating functional proteins and peptides from their membrane anchors [20]. It cleaves both membrane-bound TNF-α and its receptor TNFR1 [21, 22], and is also the major sheddase involved in EGFR-activation through release of the ligands amphiregulin, epiregulin, TGF-α and HB-EGF [23]. Amongst its many other functions, ADAM17 is thus also a key player in events governing cell survival and proliferation.

Here, we report that sphingomyelinase treatment results in PS exposure in COS7 and human endothelial cells, and this is accompanied by shedding of ADAM17 substrates. Proteolysis was suppressed in the presence of soluble phosphorylserine but not phosphorylcholine, indicating a pivotal role of PS in triggering sheddase function. Release of TNFR1 from endothelial cells concomitantly reduced the apoptotic effects of TNF-α and thus appeared to exert a protective function. Transient breakdown of membrane phospholipid asymmetry could represent a key link between the sphingolipid signaling cascade and the ADAM regulatory network.

## RESULTS

### Extracellular sphingomyelinase induces ADAM17-mediated TGF-α-AP shedding in COS7 cells and TNFR1 shedding in primary endothelial cells

**Figure 1:**
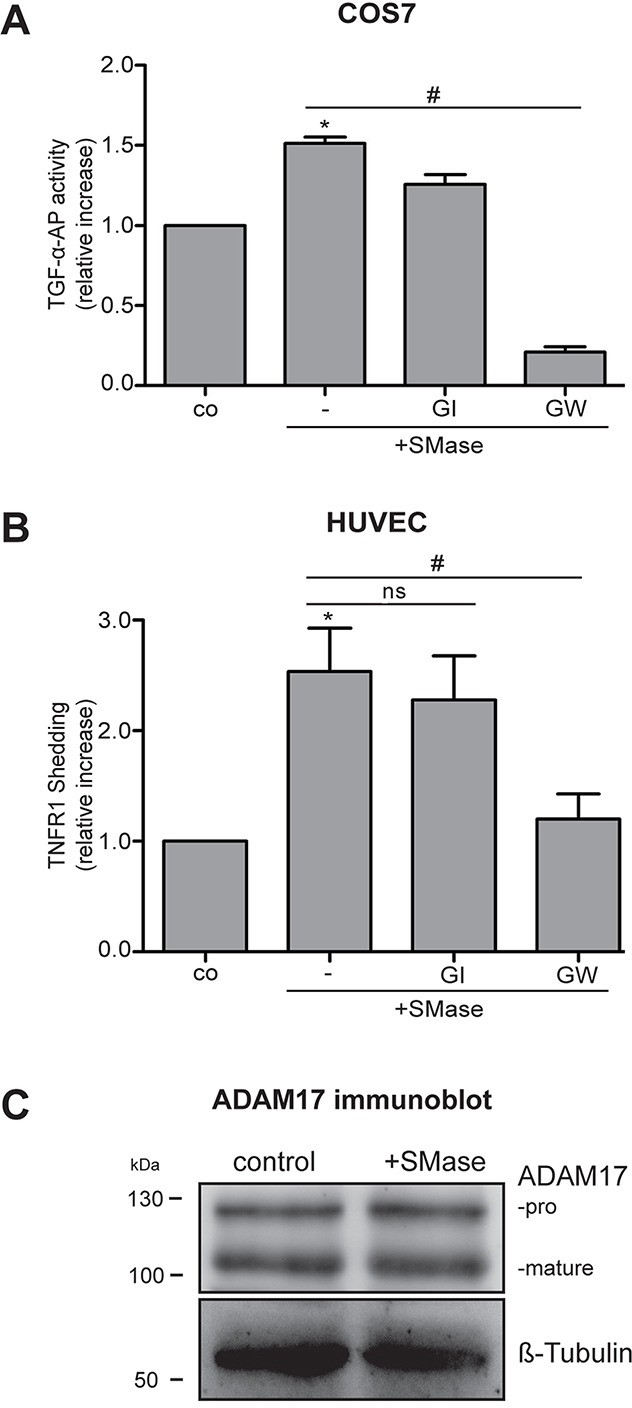
Extracellular SMase induces ADAM17-mediated shedding of TGF-α-AP in COS7 cells and TNFR1 release in HUVECs **(A)** Cells were stimulated with 0.1 U/ml SMase in the presence and absence of the ADAM10 inhibitor GI254023X (GI, 3 μM) or the ADAM17 and −10 inhibitor GW280264 (GW, 3 μM). The release of the transfected AP-tagged TGF-α was determined in COS7 cells upon 3 h of SMase treatment. **(B)** The release of the endogenous ADAM17 substrate TNFR1 in the supernatant was measured by ELISA in HUVECs after 2 h of SMase stimulation. Values are plotted as relative increase compared to control. * indicates a significant increase compared to control, # indicates a significant reduction in comparison to the stimulated sample, ns: no significant differences (*#: p<0.05; n=5; ± SEM). **(C)** HUVECs were stimulated with SMase (0.1 U/ml) for 2 h and analyzed for ADAM17 expression by immunoblot analysis. No changes in the amount of mature or pro-ADAM17 were observed. β-Tubulin was used as loading control.

ADAM17 is ubiquitously expressed in all cell types and plays various roles depending on the stimuli and available substrates. To discern whether extracellular sphingomyelinase would affect ADAM17-mediated shedding, COS7 cells were transfected with the AP-tagged ADAM17 substrate TGF-α and stimulated with 0.1 U/ml SMase for 3 hours in the presence or absence of the preferential ADAM10 inhibitor GI254023X (GI) and the ADAM10 and −17 inhibitor GW280264 (GW) [24]. The AP-activity in the supernatant and the cell lysates was determined as readout for TGF-α shedding (Figure 1A). Extracellular SMase induced the release of soluble TGF-α-AP in an ADAM17-dependent manner as indicated by the strong inhibitory effect of GW compared to GI.

Increased sphingomyelinase activity in inflammatory and infectious diseases is often observed in serum [7]. This raises the question whether endothelial cell function would be affected. Therefore, we analyzed the effect of soluble SMase on ADAM17-mediated shedding in primary human umbilical vein endothelial cells (HUVECs). These cells were treated with 0.1 U/ml SMase with or without GI or GW for 2 hours. The conditioned supernatant was then analyzed for the ADAM17 substrate TNFR1 by ELISA. Comparable to the results in COS7 cells, SMase significantly increased the release of TNFR1 into the supernatant. This was significantly inhibited by GW but not by GI (Figure 1B), indicating that ADAM17 is the responsible protease for SMase-induced TNFR1 shedding.

Regulation of ADAM17 function occurs at many levels. During ADAM17 maturation, the pro-domain is removed by pro-protein convertases [25]. Mature ADAM17 is trafficked to the cell surface to fulfill its function. To find out whether the effects of SMase on ADAM17 activation would include changes in protein expression or maturation, HUVECs were analyzed by immunoblot analysis. As shown in Figure 1C, there was no change in the total cellular amount of ADAM17 or in the maturation, clearly indicating that another mechanism must be responsible for the increased shedding capacity.

### Extracellular SMase induces phosphatidylserine exposure

We recently presented evidence that a direct interaction of ADAM17 with externalized phosphatidylserine (PS) is required for the protease to exert its sheddase function [18]. PS exposure is known to be induced by sphingomyelinase-dependent formation of ceramide [26]. Exposure to bacterial SMase was found to significantly augment phosphatidylserine exposure in erythrocytes and platelets [16, 17].

**Figure 2:**
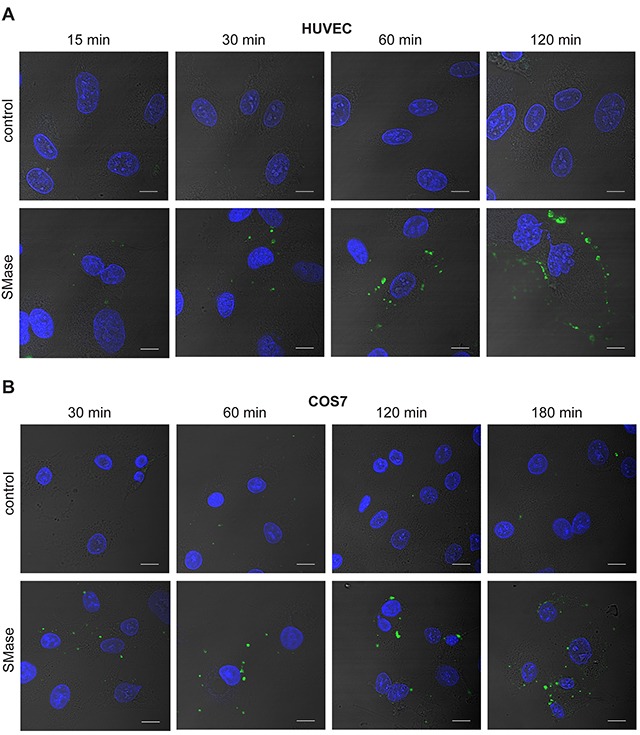
SMase stimulation induces phosphatidylserine exposure in HUVEC and COS7 cells HUVEC **(A)** and COS7 cells **(B)** were treated with 0.1 U/ml SMase for the indicated time points and stained with Annexin V-FITC for detection of externalized PS (green) and DAPI for visualizing the nucleus (blue). Representative images of three independent experiments are shown. Scale bars, 10 μm.

Whether HUVECs would respond in a similar way was addressed in the next experiment. HUVECs were stained with the fluorescently labeled PS probe Annexin V at different time points during SMase stimulation (Figure 2A). PS externalization became detectable upon 30 min and increased over the whole stimulation period. The staining was punctured and did not affect the entire plasma membrane. Although less pronounced, PS externalization could also be observed in COS7 cells in a comparable manner (Figure 2B).

### Sphingomyelinase-induced ADAM17-mediated shedding involves PS externalization

Calcium homeostasis plays a key role in the regulation of phospholipid asymmetry at the membrane by controlling the activity of scramblases and ATP-dependent flippases [27, 28].

**Figure 3:**
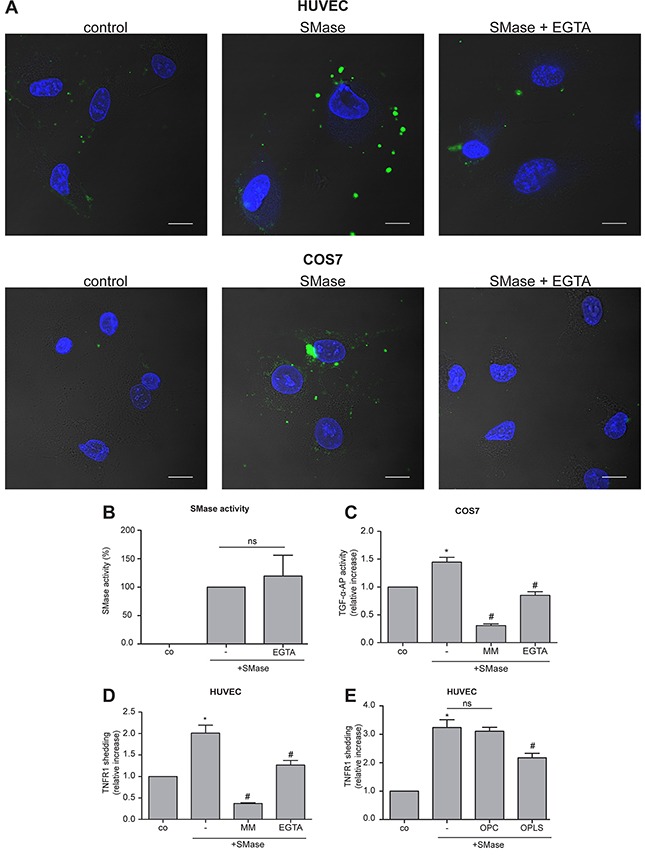
SMase-induced PS externalization and ADAM17 activation is Ca^2+^-dependent **(A)** HUVEC and COS7 cells were treated with 0.1 U/ml SMase in the presence and absence of the Ca^2+^-chelator EGTA (10 mM) for 120 or 180 min, respectively. Thereafter, cells were stained with Annexin V-FITC for detection of externalized PS (green) and DAPI for visualizing the nucleus (blue). **(B)** EGTA did not affect SMase activity. The effect of EGTA (10 mM) was controlled in a SMase activity assay. **(C, D)** SMase-induced ADAM17-mediated shedding was analyzed in COS7 cells and HUVECs as stated in Figure 1 in the presence or absence of the metalloprotease inhibitor marimastat (MM, 10 μM) or the Ca^2+^-chelator EGTA (10 mM). **(E)** SMase-induced TNFR1 shedding was significantly reduced by addition of the competing phosphatidylserine head group (OPLS) but not by the head group of PC (OPC). * indicates a significant increase compared to control, # indicates a significant reduction in comparison to the stimulated sample, ns: no significant differences (*#: p<0.05; n=5; ± SEM).

Non-apoptotic, transient PS exposure is often mediated by Ca^2+^-dependent phospholipid scramblases including the transmembrane protein 16F (TMEM16F), also called Anoctamin-6 (ANO6) [29, 30]. ANO6 activity strongly depends on the presence of Ca^2+^ [29]. Based on this fact, we investigated whether Ca^2+^ might play a role in the SMase-induced PS scrambling. Indeed, the Ca^2+^-chelator EGTA markedly reduced phospholipid externalization in COS7 cells and in HUVECs (Figure 3A) at any time point without affecting the SMase activity itself (Figure 3B). To test whether this reduced PS exposure would be accompanied by reduced ADAM17 activity, both cell types were analyzed in parallel in shedding assays. Indeed, SMase-induced ADAM17-mediated shedding was significantly diminished upon EGTA treatment (Figure 3C and 3D). Control experiments using the classical ADAM17 activator phorbol-myristate-acetate (PMA), that also acts via PS exposure in keratinocytes [18], were performed (Supplementary Figure 1). Indeed, EGTA also reduced PMA-stimulated PS exposure and concomitantly shedding in COS7 cells. To exclude that EGTA just extracted Zn^2+^ that was required for ADAM activity cells were pre-incubated with EGTA and washed before PMA stimulation. No differences in shedding induction were observed, leading to the conclusion that EGTA does not affect the *bona fide* catalytic activity of ADAM17.

To directly probe the relevance of externalized PS, HUVECs were treated with SMase in the presence or absence of OPLS or OPC, the soluble head group of phosphatidylserine or phosphatidylcholine, respectively. Whereas OPC did not affect SMase-induced TNFR1 shedding, OPLS led to a substantial reduction of substrate release (Figure 3E).

### TNFR1 shedding in HUVECs decreases TNF-α sensitivity

**Figure 4:**
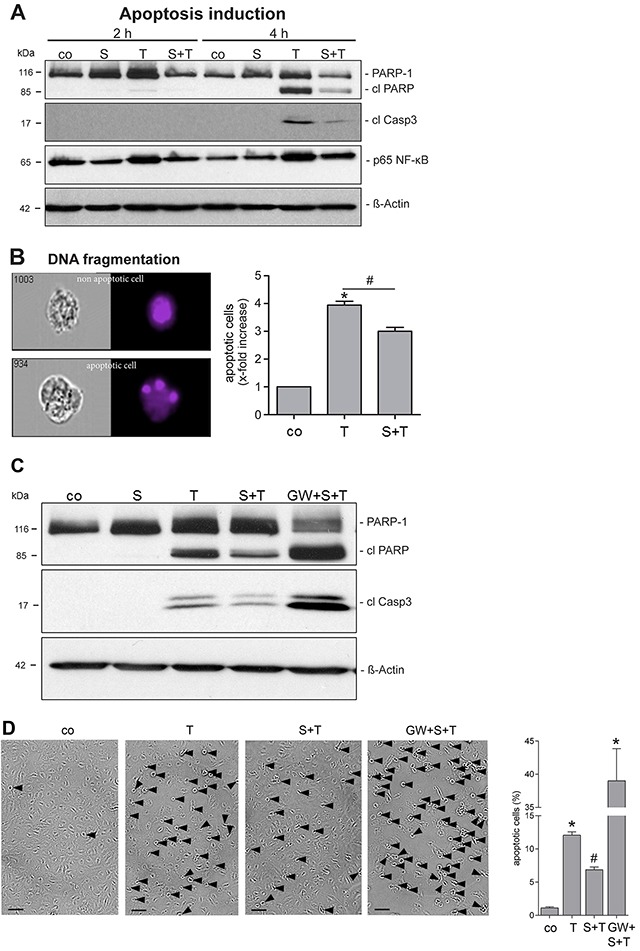
SMase treatment reduces the TNF-α sensitivity of endothelial cells **(A)** HUVECs were pretreated with SMase (S, 0.1 U/ml, 2 h) and stimulated with TNF-α (T, 40 ng/ml + CHX (5 μg/ml) for 2 or 4 h, followed by lysis and Western blot analyses. Shown are representative Western blots for total PARP-1, cleaved PARP (cl PARP), cleaved caspase-3 (cl Casp3), and phosphorylated p65 NF-κB. β-Actin served as a loading control. **(B)** Apoptosis was additionally measured by quantifying nuclear fragmentation by ImageStream analysis. HUVECs were analyzed after overnight incubation with TNF-α/CHX with or without SMase pretreatment as described. The means of three different independent experiments are shown. * indicates a significant increase compared to control, # indicates a significant reduction in comparison to the stimulated sample (*#: p<0.05). Data are represented as mean ± SEM. **(C, D)** TNF-α-induced cell death is reduced upon SMase pre-treatment and reinforced upon ADAM17 inhibition. HUVECs were pretreated with SMase (SM, 0.1 U/ml, 2 h) in the presence or absence of the inhibitor GW (3 μM) and stimulated with TNF-α (T, 40 ng/ml) + CHX (5 μg/ml) for 4h. (C) Cells were analyzed by Western blotting. Shown is a representative blot (n=4) for cleaved PARP-1 (cl PARP), and cleaved caspase-3 (cl Casp3), β-Actin served as a loading control. (D) The number of HUVECs showing TNF-α-induced cell membrane blebbing is reduced upon SMase pre-treatment and reinforced upon ADAM17 inhibition. Cells were stimulated as described above and photographs were taken. Scale bars, 50 μm. The percentage of cells was determined by counting blebbing cells compared to the number of total cells on 3 randomly selected fields. * indicates a significant increase compared to control, # indicates a significant reduction in comparison to the TNF-α-stimulated sample (*#: p<0.05; n=6; ± SEM; one-way ANOVA with post-hoc Tukey test).

Binding of TNF-α to the TNFR1 on endothelial cells is followed by rapid activation of the transcription factor NF-κB. Initiator caspases cleave effector caspases including caspase-3. Caspase activation leads to cleavage of poly(ADP-ribose) polymerase-1 (PARP-1) and to the characteristic membrane blebbing, DNA fragmentation and laddering associated with apoptotic death. To analyze the SMase effect on TNF-α-induced cell death, HUVECs were pretreated with SMase following incubation with TNF-α. The level of activated p65 NF-κB, cleaved caspase-3 (cl Casp3), and the generation of cleaved PARP-1 (cl PARP) were explored after 2 and 4 hours by immunoblot analysis. Despite equal protein loading, the amount of total PARP-1 protein showed some variability, that was, however, not significant (Supplementary Figure 2). The TNF-α-induced increase of p65 NF-κB, cl caspase-3, and cl PARP was clearly visible after 4 hours (Figure 4A). Pre-incubation with SMase reduced the induction of the apoptotic cascade.

The protective effect of SMase on the apoptotic response was additionally measured by nuclear fragmentation analysis using ImageStream cytometry. As to be expected, HUVECs showed strong nuclear fragmentation after TNF-α exposure (Figure 4B). This effect was significantly reduced when cells were pretreated with SMase. To ensure that a direct link between SMase and ADAM17 activation exists, cells were pretreated with the GW inhibitor and analyzed for TNF-α-induced cell death by immunoblot (Figure 4C). Indeed, inhibition of ADAM17 abrogated the protective SMase effect. Generation of cleaved PARP-1 and caspase-3 was much stronger in the presence of GW compared to only TNF-α treated cells. The protective role of external SMase as well as the opposite effect of GW was also indicated by corresponding changes in the number of blebbing cells induced by TNF-α incubation (Figure 4D and Supplementary Figure 3). Overall, these results show that SMase-induced ADAM17 activation counteracts TNF-α-induced cell death of endothelial cells.

## DISCUSSION

SMases plays a central role in the production of ceramide, a bioactive molecule with contradictory properties. Ceramide generation by cellular SMases accompanies apoptosis, and a role for the molecule in the events leading to cell death is being discussed. On the other hand, generation of ceramide in the absence of concomitant triggering events is not cytotoxic. Thus, endothelial cells, fibroblasts and resting lymphocytes tolerate treatment with high doses of bacterial SMase with no loss of viability [31].

Further to its roles as a signaling molecule, ceramide affects the microarchitecture of cell membranes, directing lateral flow of lipids and proteins to orchestrate the function of signaling platforms and regulate membrane movement [7, 32–34]. In glial cells, ceramide may assume a prime function in promoting formation of microvesicles [35, 36], an aspect that deserves to be followed up in other cell systems including endothelial cells.

Moreover, ceramide generated in erythrocytes and platelets through treatment of cells with bacterial SMase provokes breakdown of membrane phospholipid asymmetry and exposure of PS at the external membrane face. We anticipated that the latter phenomenon would extend to nucleated cells, in which case a novel link could emerge between ceramide and the regulatory network of membrane-anchored sheddases. ADAM17, eminent member of this family, assumes vitally important functions through liberation of biologically active substrates from cell surfaces. ADAM17 sheddase function is induced by a wide variety of agents [23]. A long-standing enigma relates to how signals generated along different pathways within the cell can be relayed to one and the same protease that is oriented at the cell surface. Recently, we presented evidence that PS exposure was the solution to this problem. Classical ADAM17 activation pathways were all found to provoke transient PS-translocation to the outer membrane leaflet. A cationic motif was identified in the membrane-proximal domain of ADAM17 that specifically interacted with phosphorylserine. Evidence was obtained that through this interaction, the protease would be attracted, enabling the catalytic domain to find its substrate. The model at the same time provided a simple explanation for the known fact that shedding of ADAM17 substrates accompanies apoptosis [18].

Although it is well known that SMase activity is elevated in the serum of patients suffering from several diseases [7], it is not clear where and how secreted SMase is active. This makes it impossible to compare the activities of exogenously applied SMase in cell culture experiments to endogenously secreted SMase in serum. Here, we used SMase concentrations of 0.1 U/ml. This concentration is in the range of SMase activity shown to induce ceramide-rich membrane regions in Molt-4 cells without inducing apoptosis [37], below the concentration that induces shedding of L-Selectin in granulocytes [31] and in the range of SMase activity shown to modify LDL particle *in vitro* [38]. It would be of interest to evaluate the role of endogenous SMase that is secreted upon stimulation with inflammatory mediators (TNF-α, Interferon-gamma) [6] for ADAM17 activation in the future.

Here, we took the lead provided by work on erythrocytes and platelets and examined the effects of exogenously applied SMase treatment on PS exposure in nucleated cells. As expected from the literature, COS7 and HUVECs withstood treatment with the enzyme without loss of viability, confirming that ceramide itself is not cytotoxic. But its generation did produce dramatic effects on membrane architecture. PS exposure became evident after 30 min exposure of cells to soluble SMase and increased steadily thereafter. PS exposure was temporally linked to upregulation of ADAM17 function. This was shown with the use of two established model systems: first, the cleavage of AP-tagged TGF-α in transfected COS7 cells and second, the release of endogenous TNFR1 from HUVECs. Both models yielded the same results and, through employment of the previous strategy, PS externalization could be linked to enhanced substrate shedding. Thus, shedding was significantly inhibited in the presence of competing, soluble phosphorylserine, but not by phosphorylcholine, in the face of unaltered cell-surface expression of ADAM17. The asymmetric distribution of phospholipids is mediated by various transporters: PS and PE passively translocating to the external leaflet are normally returned to the inner leaflet through the action of ATP-dependent “flippases”. Upon activation, scramblases non-specifically and bidirectionally translocate phospholipids between the outer and inner leaflets of the plasma membrane [39]. Function of the recently identified scramblase Anoctamin-6 depends on calcium [29, 40]. This will most likely be the case for other scramblases as well. Thus, the finding that EGTA suppressed both PS externalization and TNFR1-release provided another line of support for our assumption.

In the context of cell death or survival, the net effect of ADAM17 sheddase activation may be expected to vary dependent on cell types with their respective expression of shed death ligands, death receptors and growth factors. Exploratory experiments were performed with endothelial cells for two reasons. First, TNF-α-mediated endothelial cell death is a phenomenon of widespread importance. The inflammatory cytokine is involved in the development of atherosclerosis, reperfusion injury, hypertrophy and heart failure [41]. Second, among the shed ADAM17 substrates in endothelial cells, TNFR1 predominates over TNF-α and growth factors, which facilitates interpretation of the experimental outcome. A clear protective effect was observed. Thus, shedding of TNFR1 effected by pretreatment with sphingomyelinase blunted subsequent TNF-α-induced apoptosis. This was reflected by a marked reduction in cleaved caspase-3 and cleaved PARP a few hours after TNF-α treatment. The protective effect of SMase was reflected in conspicuous reduction of nuclear fragmentation. Incubation with the inhibitor GW abrogated these effects, providing a clear link between SMase and ADAM17 activation. The results tie in with the general concept that cleaved TNFR1 can bind away and thus protect neighboring cells from the action of TNF-α [42–45]. GW treatment appeared to even exacerbate cell death, highlighting the vital importance of ADAM17. The additional effect might be due to several factors. ADAM17 displays constitutive activity; this might cause protective effects to accumulate over time. These would be inhibited by GW. Moreover, TNF-α induces reportedly the release of endogenous SMase in endothelial cells [6], that might act in addition to the extracellularly applied enzyme. In this context, the evaluation of the role of endogenous SMase on ADAM17-mediated TNFR1 shedding becomes of high interest. Moreover, TNF-α is known to induce several chemokines and adhesion molecules in endothelial cells and this may also have proatherogenic effects. The question would follow whether these inflammatory responses would be modified by GW, ADAM17 knockdown or by targeting endogenous SMase. It would be of high interest to address such questions in future studies.

**Figure 5:**
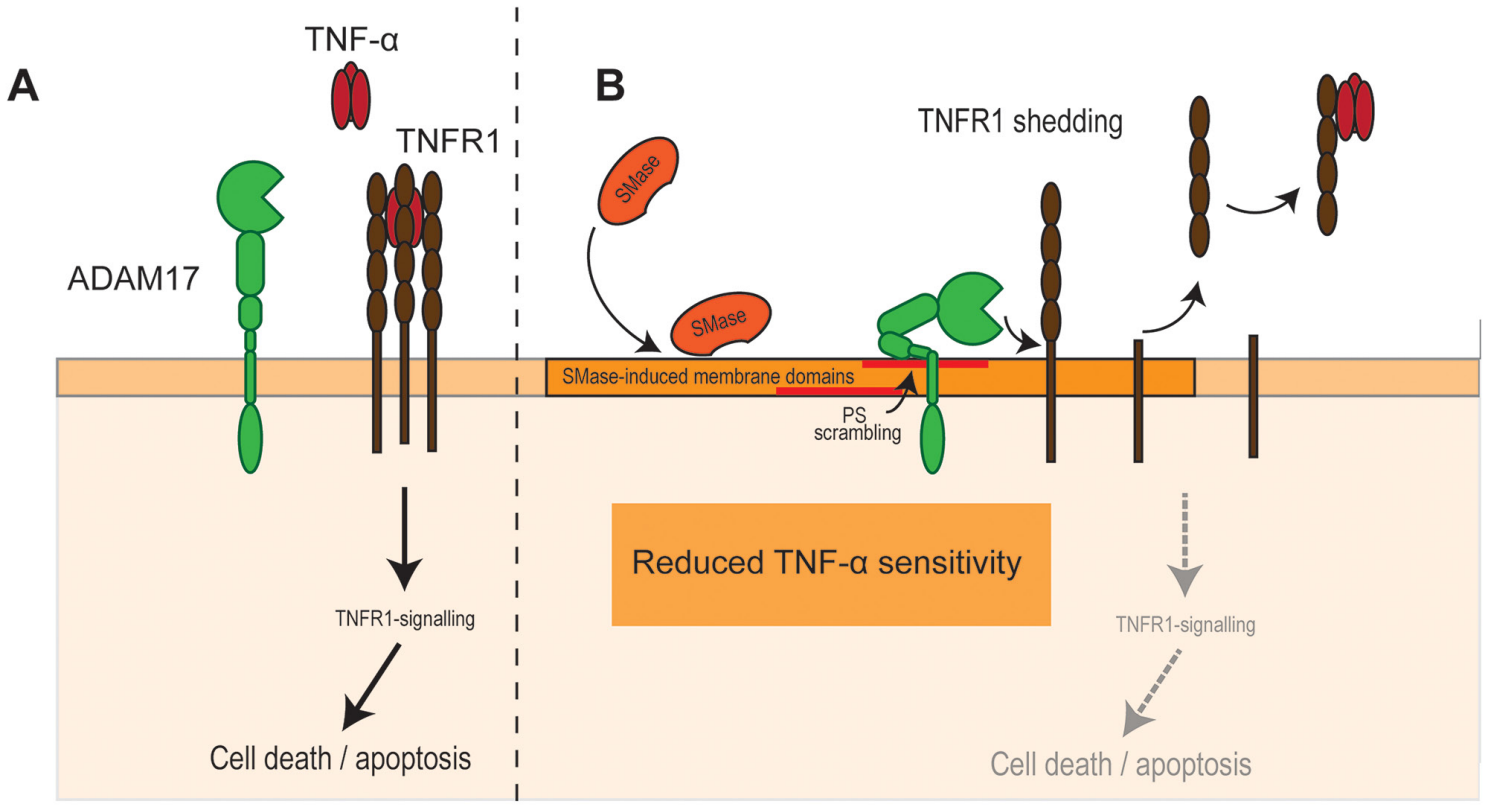
SMase activity impairs TNF-α-induced cell death in endothelial cells **(A)** TNF-α is a major trigger of endothelial apoptosis. **(B)** SMase activity leads to cell membrane changes and PS externalization from the inner to the outer cell membrane leaflet. PS-interaction enables ADAM17 to release its substrate TNFR1 thus impairing TNF-α-induced cell death.

Based on our data presented here, we propose the following model (Figure 5). SMase activity leads to cell membrane changes and PS externalization from the inner to the outer cell membrane leaflet. In endothelial cells, PS-interaction enables ADAM17 to release its substrate TNFR1 thus impairing TNF-α-induced apoptosis. In other cell types, different consequences might follow.

In conjunction with previous investigations, a possible circle of events now lends itself to speculation regarding the possible significance of ceramide generation in apoptosis. Existing evidence indicates that breakdown of phospholipid asymmetry during induced apoptosis can entail not only the translocation of PS to the outer membrane leaflet, but also a concomitant movement of sphingomyelin to the inner leaflet, where the molecule is cleaved by nSMase. The ceramide thus generated was not directly involved in the death cascade, but was instrumental in promoting membrane re-organization and blebbing [10]. Two aspects may now be added to the scenario. First, generation of ceramide will itself promote breakdown of phospholipid asymmetry, and a self-perpetuating circle of events may thus arise. Second, ADAM17 upregulation by externalized PS may serve a protective function. In endothelial cells, this may be effected mainly be the release of decoy TNFR1. In others, liberation of membrane-anchored growth factors, in particular EGFR-ligands, may play relevant roles.

In addition, several other aspects should be considered: Pore-forming bacterial toxins perturb membrane organization and this has been shown to promote ceramide formation via nSMase, which in turn leads to ADAM17 activation [14, 31]. Deficiency of nSMase activity blunts the capacity of *S. aureus* to invoke pulmonary damage, which is dependent on cleavage of syndecan1 by ADAM17 [46, 47].

Taken together, our results demonstrate a hitherto undescribed link between sphingomyelinase activity and ADAM17 function arising from breakdown of phospholipid asymmetry. Depending on the cell type and the substrates, SMase-induced ADAM17 activation may lead to multifaceted functional consequences. Our data on endothelial cells clearly support the view that ADAM17-mediated TNFR1 shedding counteracts TNF-α-induced cell death. Overall, many other connections between ceramide formation and the ADAM-regulatory network are likely to be uncovered through directed searches in the foreseeable future.

## MATERIALS AND METHODS

### Reagents

Sphingomyelinase from *Staphylococcus aureus*, Annexin V-FITC, cycloheximide (CHX), O-phospho-L-serine (OPLS), phosphorylcholine (OPC), phorbol 12-myristate-13-acetate (PMA), and EGTA (ethylene glycol tetraacetic acid) were obtained from Sigma. OPLS and OPC were dissolved in H_2_O and adjusted to pH 7 before use. Hydroxamate-based ADAM17/10 inhibitor GW280264 [24] was purchased from Aeobious. Marimastat and ADAM10 inhibitor GI254023X [24] were obtained from Tocris Bioscience. Highly purified human recombinant TNF-α was provided by BASF Bioresearch.

### Cell culture

COS7 cells (ATCC) were grown in high glucose Dulbecco's Modified Eagle Medium (DMEM) supplemented with 10% (v/v) heat inactivated fetal calf serum, 100 U/ml penicillin, and 100 μg/ml streptomycin (P/S). Human umbilical vein endothelial cells (HUVECs) from multiple donors were purchased from PromoCell and grown in endothelial cell growth medium (PromoCell) and 1% P/S. Passages between two and eight were used for experiments.

### Transfection and TGF-α-AP shedding assay

The plasmid for alkaline phosphatase tagged TGF-α expression was from Carl Blobel (Hospital for Special Surgery, New York, USA). COS7 cells were transfected with Turbofect Transfection Reagent (Thermo Fisher Scientific) according to the manufacturer's instructions. 24 h after transfection medium was replaced by fresh DMEM and pre-incubated for 1 h before stimulation with sphingomyelinase. Supernatants and cell lysates (lysed in 2.5% (v/v) Triton-X in H_2_O with 1 mM EDTA and 10 mM 1,10-phenantroline) were collected and the AP-activity was determined by employing the AP-substrate 4-nitrophenyl phosphate (Sigma Aldrich, 1 mg/ml). For shedding analyses, the relative AP-activity of supernatant compared to the total AP-activity of supernatant plus cell lysates normalized to the unstimulated control is shown.

### TNFR1 enzyme linked immunosorbent assay

TNFR1 ELISA was obtained from R&D Systems and performed according to manufacturer's instructions. HUVECs were grown to 90% confluence in 12-well plates in endothelial growth medium. Before stimulation, medium was changed to endothelial basal medium and pre-incubation with or without the corresponding inhibitors took place for 15 to 30 min. Thereafter, 0.1 U/ml sphingomyelinase was added for 2 h. The resulting supernatants were cleared by centrifugation and stored at −20°C until the ELISA were performed.

### Annexin V-FITC staining

Cells were grown on coverslips (collagenated for HUVECs) to semi-confluence. After the indicated stimulation/inhibition periods the coverslips were incubated with a 1:20 solution of Annexin V-FITC in Annexin-Binding-Buffer (ABB; 10 mM HEPES, 140 mM NaCl, 2.5 mM CaCl_2_, pH 7.4) for 5 min in the dark at room temperature. The coverslips were carefully washed twice with ABB and fixed with 3% (v/v) paraformaldehyde for 15 min. Then, the coverslips were washed six times with PBS, once with distilled water and mounted in Mowiol embedding medium containing DABCO and DAPI.

### Immunoblot analysis

For analysing TNF-α-induced cell signaling, cells were lysed at 4°C in TNE buffer (50 mM Tris, pH 8.0; 1% (v/v) NP-40, 150 mM NaCl, 3 mM EDTA, 1 mM sodium orthovanadate, 5 mM sodium fluoride, 20 μM z-VAD-FMK, and cOmplete inhibitor cocktail (Roche)). Identical amounts of protein per lane were resolved by electrophoresis on SDS-polyacrylamide gels and transferred onto nitrocellulose membranes by Western blotting. Proteins were detected by using antibodies specific for p65 NF-κB (Cell Signaling, catalogue number 3033S), PARP-1 (Cell Signaling, catalogue number 9542), cleaved caspase-3 (Cell Signaling, catalogue number 9661 L/S) and were stained with the corresponding peroxidase-coupled secondary antibody (Jackson ImmunoResearch, catalogue number 111-035-146 and 111-035-146) and the LumiGLO chemiluminescent substrate (Cell Signaling, Danvers, MA) and captured on Amersham Hyperfilm ECL (GE Healthcare, Munich, Germany).

For analysing ADAM17 expression, cells were washed once with PBS and lysed in lysis buffer (5 mM Tris-HCl pH 7.5, 1 mM EGTA, 250 mM saccharose and 1% (v/v) Triton X-100) supplemented with cOmplete inhibitor cocktail (Roche Applied Science) and 10 mM 1,10-phenantroline. Equal amounts of protein were loaded on 10% SDS–PAGE gels. The samples were electrotransferred onto polyvinylidene difluoride membranes (Hybond-P; Amersham) and blocked for 1 h with 5% skim milk in Tris-buffered saline containing 0.1% Tween-20 (TBST). After 1 h incubation with the anti-ADAM17 antibody (Chemicon AB19027, 1:2000) in TBST, the membranes were washed three times in TBST. The primary antibody was detected using affinity-purified peroxidase (POD)-conjugated secondary antibodies (1:10000) for 1 h at room temperature. Detection was carried out using the ECL detection system (Amersham). Signals were recorded by a luminescent image analyzer (Fusion FX7 imaging system; PEQLAB Biotechnologie). Equal loading as well as efficiency of transfer were routinely verified for all Western blots by reprobing the membranes for Actin or Tubulin (Sigma, catalogue number A1978; DSHB clone E7).

### Sphingomyelinase activity assay

The fluorometric sphingomyelinase activity assay (Cayman Chemicals, no. 10006964) was used to analyze the relative SMase activity. In brief, 0.1 U/ml bacterial SMase was added to endothelial basal medium with or without 10 mM EGTA and the SMase activity was determined according to manufacturer's instructions.

### Image acquisition and processing

Image acquisition of Annexin V-FITC stained cells was performed with an inverted confocal microscope (Fluoview FV1000, Olympus) using a UPLSAPO 60 oil-immersion objective (numerical aperture: 1.35) and 2x zoom. Annexin V–FITC was excited at 488 nm and emission was recorded at 520 nm. Images were acquired with the same laser and detection settings for each experimental setup. Phase contrast images for quantification of blebbing cells were taken with a Canon DS126291 camera and a Hund Wetzlar Wllovert30 microscope. Calculation was performed by counting blebbing cells in 3 random microscope fields of six cover slips from independent experiments, respectively, and expressing these counts as a proportion of total cells in identical fields.

### Imagestream apoptosis assays

The percentage of cells with fragmented DNA was measured with the ImageStream (Amnis/EMD Millipore) system as described in [48]. For that, two 10 cm tissue flasks per sample of semiconfluent HUVECs were stimulated as indicated, detached with Accutase® (Thermo Fisher Scientific), washed in PBS with 2 mM EDTA and stained with Hoechst (dilution 1:10.000) for 30 min on ice. The apoptosis wizard plug-in was used for assaying the number of cells showing nuclear fragmentation compared to cells with intact nuclei.

### Statistical analysis

If not otherwise indicated, One-Way ANOVA and subsequent Bonferroni post-tests were performed.

## SUPPLEMENTARY MATERIALS FIGURES



## Abbreviations

ADAM: a disintegrin and metalloprotease
ANO6: Anoctamin-6
AP: alkaline-phosphatase
aSMase: acid sphingomyelinase
ATP: adenosine triphosphate
CHX: cycloheximide
EGFR: epithelial growth factor receptor
HB-EGF: heparin binding–epithelial growth factor
HUVECs: human umbilical vein endothelial cells
NF-kB: nuclear factor kappa-light-chain-enhancer of activated B cells
nSMase: neutral sphingomyelinase
OPC: phosphorylcholine
OPLS: O-phospho-L-serine
PARP-1: poly[ADP-Ribose] polymerase 1
PS: phosphatidylserine
PMA: phorbol 12-myristate 13-acetate
SMase: sphingomyelinase
TGF-α: transforming growth factor-alpha
TMEM16F: transmembrane protein 16F
TNF-α: tumor necrosis factor-alpha
TNFR1: tumor necrosis factor-alpha receptor 1.
