# Supplementary material for: Extracellular sphingomyelinase activity impairs TNF-α-induced endothelial cell death via ADAM17 activation and TNF receptor 1 shedding
Source: Oncotarget. 2017 Aug 7;8(42):72584–96. doi: 10.18632/oncotarget.19983 (PMC5641154; doi:10.18632/oncotarget.19983)
Supplement: Supplementary file 1 [file oncotarget-08-72584-s001.pdf]

Extracellular sphingomyelinase activity impairs TNF-α-induced endothelial cell death via ADAM17 activation and TNF receptor 1 shedding

SUPPLEMENTARY MATERIALS

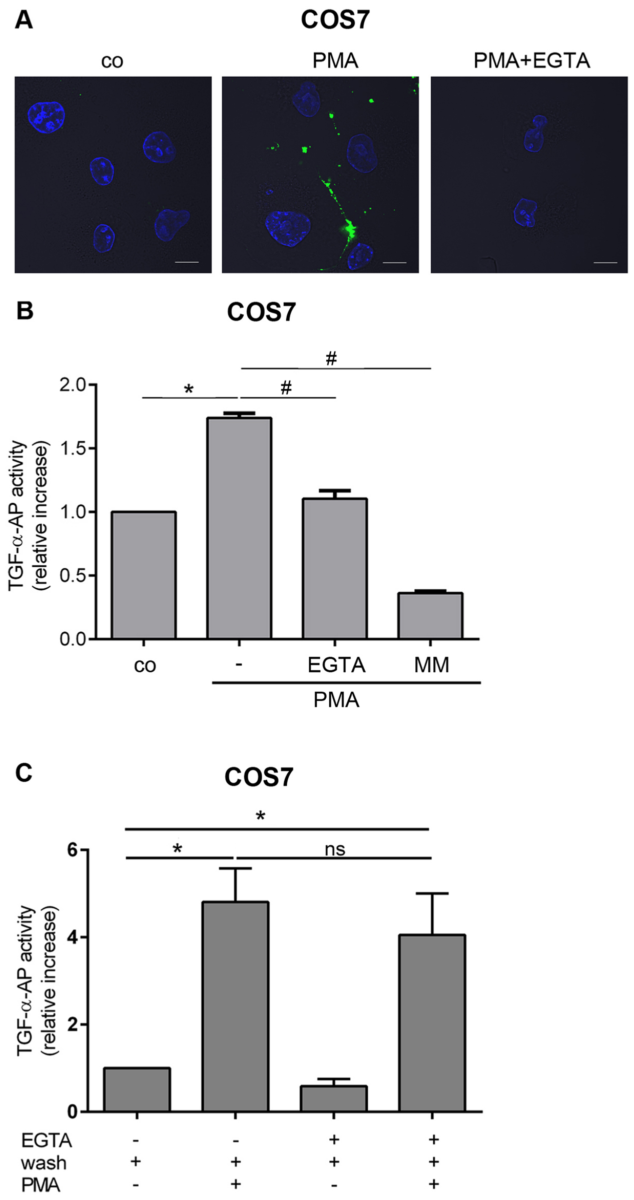

Supplementary Figure 1: (A) COS7 cells were stimulated with PMA (200 ng/ml) in the presence or absence of EGTA (10 mM) for 60 min and analyzed for PS exposure via Annexin-V staining. Scale bars: 10 µm. **(B)** COS7 cells were stimulated as described in the presence or absence of EGTA (10 mM) or marimastat (MM, 10 µM) and analyzed for release of the transfected AP-tagged TGF-α. **(C)** COS7 cells were incubated with EGTA (10 mM) for 30 min and washed 3 times with PBS. Thereafter, cells were stimulated with PMA as described and shedding of AP-tagged TGF-α was quantified. * indicates a significant increase compared to control. # indicates a significant reduction in comparison to the stimulated sample (*/#: $p<0.05$). ns = no significant differences. Data are represented as mean ± SEM (n=3).

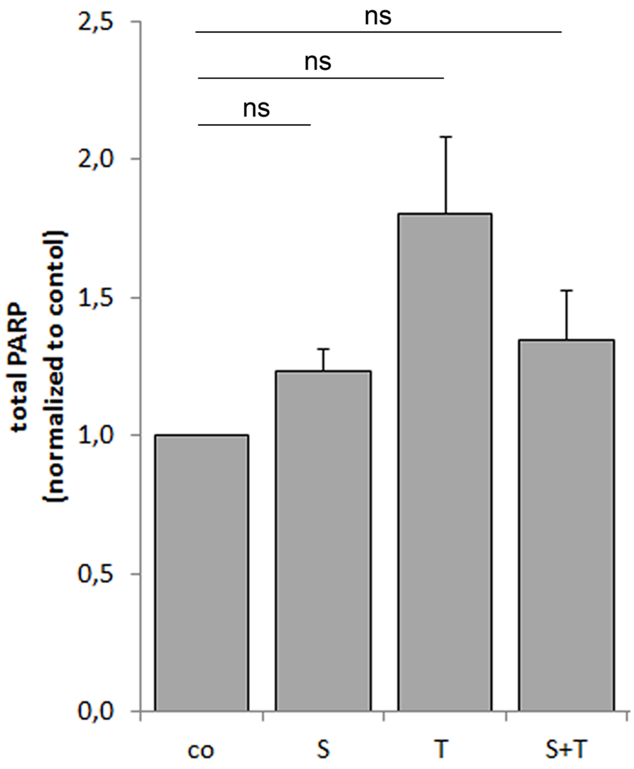

Supplementary Figure 2: Quantification of total PARP-1. HUVECs were pretreated with SMase (S, 0.1 U/ml, 2 h) and stimulated with TNF-α (T, 40 ng/ml + CHX (5 µg/ml) for 4 h, followed by lysis and Western blot analyses. Densitometric analysis of total PARP-1 protein (cleaved PARP plus full length PARP) of 4 independent experiments. No significant differences were observed. ns = no significant differences. Data are represented as mean ± SEM (n=4).

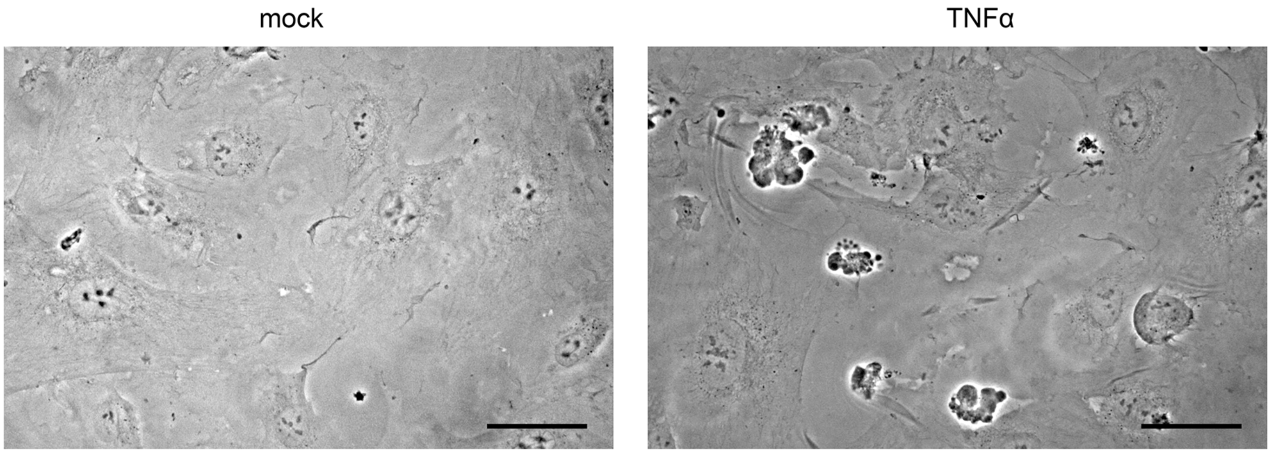

Supplementary Figure 3: Images of TNF-α-induced cell membrane blebbing. HUVECs were stimulated with TNF-α (T, 40 ng/ml) + CHX (5 µg/ml) for 4h and photographs were taken. Scale bars, 20 µm.
